# Excess mortality in a cohort of Brazilian patients with a median follow-up of 11 years after the first psychiatric hospital admission

**DOI:** 10.1007/s00127-022-02304-z

**Published:** 2022-05-31

**Authors:** Daiane Leite da Roza, Marcos Gonçalves de Rezende, Régis Eric Maia Barros, João Mazzoncini de Azevedo-Marques, Jair Lício Ferreira Santos, Lilian Cristina Correia Morais, Carlos Eugenio de Carvalho Ferreira, Bernadette Cunha Waldvogel, Paulo Rossi Menezes, Cristina Marta Del-Ben

**Affiliations:** 1 Division of Psychiatry, Department of Neurosciences and Behavior, Ribeirão Preto Medical School, University of São Paulo, Bandeirantes Avenue, 3900, Ribeirão Preto - SP, 14049-900 Brazil; 2 Department of Social Medicine, Ribeirão Preto Medical School, University of São Paulo, Ribeirão Preto - SP, Brazil; 3 Fundação Sistema Estadual de Análise de Dados, SEADE, São Paulo - SP, Brazil; 4 Department of Preventive Medicine, Faculty of Medicine, University of São Paulo, São Paulo - SP, Brazil; 5 Population Mental Health Research Centre, University of São Paulo, São Paulo - SP, Brazil

**Keywords:** Mortality, Mental disorders, Low‐ and middle‐income countries, Years of life lost, Causes of death

## Abstract

**Purpose:**

To estimate the mortality rates of a cohort of Brazilian patients after their first psychiatric admission and determine the possible risk factors associated with excess mortality.

**Methods:**

The study included a cohort of psychiatric patients hospitalised from Jan 1, 2002 to Dec 31, 2007 in the catchment area of Ribeirão Preto, São Paulo state, Brazil. Data were linked to deaths that occurred between Jan 1, 2002 and Dec 31, 2016 from the SEADE Foundation (state data analysis system of São Paulo). The mortality rate (MR), age-sex-standardised mortality ratio (SMR), life expectancy at birth, and years of life lost (YLL) were computed. The factors associated with mortality were analysed by survival analysis using a Cox proportional hazards regression model.

**Results:**

Of 4019 patients admitted (54.76% male), 803 died (69.74% male) during the follow-up (median = 11.25 years). Mortality rates were approximately three-fold higher than expected (SMR = 2.90, 95% CI 2.71–3.11). The highest mortality rate was noted in men with alcohol-related disorders (SMR = 5.50, 95% CI 4.87–6.19). Male sex (adjusted hazard ratio (aHR) = 1.62, 95% CI 1.37–1.92), higher age (aHR = 21.47, 95% CI 13.48–34.17), and unemployment (aHR = 1.22, 95% CI 1.05–1.43) significantly increased the mortality risk from all causes. The average YLL was 27.64 years with the highest YLL noted in nonalcohol substance-related disorders (39.22 years). The life expectancy at birth in this cohort was 47.27 years. Unnatural causes of death were associated with nonwhite skin colour and substance-related disorders.

**Conclusion:**

An excess of mortality and a significant reduction in life expectancy of mentally disordered patients who were first admitted to psychiatric beds was noted, particularly patients admitted for substance-related disorders, which should represent a priority in mental health policies.

**Supplementary Information:**

The online version contains supplementary material available at 10.1007/s00127-022-02304-z.

## Introduction

People with mental disorders are more likely to die prematurely than the general population with an estimated relative risk (RR) of 2.22 (95% CI 2.12–2.33) and 10 years of life lost according to a meta-analysis [1] that included studies from 29 countries.

The risk is not similar for all mental health problems, and the underlying causes of death can also vary [2, 3]. Individuals suffering from severe mental disorders, especially psychotic disorders [4], depressive disorders [5], and comorbid substance use disorders [6], are at higher risk of mortality and significantly reduced life expectancy [7], and approximately 20 years of life is lost [8, 9].

In addition, patients who have undergone psychiatric hospitalisation are generally more seriously ill individuals and, therefore, have an increased risk of premature mortality [10], especially deaths from suicide [11]. Several studies that integrate a recent meta-analysis [10] indicate that the risk of mortality is especially high soon after discharge from psychiatric hospitalisation.

The excess mortality in mental disorders is currently well documented for high-income countries (HICs), mainly in Europe and North America. Of the 203 studies included in the meta-analysis cited above [1], only ten were from low- and middle-income countries (LMICs), of which only one was from Brazil. Information regarding the health consequences of mental disorders in LMICs is relevant given that these countries, predominantly located in Africa, Asia, and Latin America, represent more than 85% of the world’s population [12] and are responsible for almost all premature deaths (30 to 69 years) from noncommunicable diseases (NCDs) that occur worldwide [13].

The few studies about the mortality rates in mental disorders conducted in LMICs, contradict the belief that the evolution and prognosis of psychoses would be more benign in LMICs than in HICs [14]. Evidence suggests that the association between mental disorders and mortality in LMICs may be similar to or even higher than those estimated for HICs. For instance, studies performed in Ethiopia have reported a standardised mortality ratio (SMR) between 2.14 (95% CI 1.77–2.56) [15] and 5.98 (95% CI 4.09–7.87) [16]. In Asia, the SMR varied from 1.62 (95% CI 1.57–1.68) [17] in Korea to 6.80 (95% CI 6.30–7.40) [18] in Central Asia. The only study in Latin America with Brazilian patients admitted to a psychiatric hospital showed mortality rates (SMR = 8.40, 95% CI 4.00–15.90) [19] greater than those registered in other LMICS.

Many changes have occurred in the Brazilian mental health policies since the 1990s, with the definitive implementation of the Unified Health System (SUS) and the reform of the psychiatric system when Brazil started to follow international guidelines for treatment and offer free medical care to all Brazilians. However, the lack of current data reduces the visibility of premature mortality in mental disorders and hampers the defence of its inclusion as a priority in health care initiatives.

Given the relevance of updated estimates for countries with significant social inequalities and severe economic constraints, we aimed to estimate the mortality rates in individuals from a cohort of Brazilian patients after their first psychiatric admission and to determine possible risk factors associated with excess mortality and the causes of premature death. We hypothesised that our patients would have higher mortality rates and lose more years of life than patients from HICs.

## Methods

### Context of the study

This study was conducted in the Ribeirão Preto catchment area, located in the state of São Paulo, Brazil. The state of São Paulo comprises 645 municipalities and is the most developed state in Brazil, accounting for 31% of the country's gross domestic product (GDP). Life expectancy at birth of individuals in the state of São Paulo (75.04 years) was approximately 2 years greater than the average for Brazil (73.48 years) according to the last Brazilian Census of 2010 (https://cidades.ibge.gov.br/). The Ribeirão Preto catchment area comprises 26 municipalities in a territorial area of 10,852 km^2^ with 1,328,535 inhabitants and a population density of 122.42 inhabitants/km^2^. The Human Development Index (HDI) for this region ranged from 0.69 to 0.80. Ribeirão Preto is the main municipality of the region with 604,682 inhabitants. The median population of the remaining 25 municipalities was 23,871 (range from 1953 to 110,094), and the region’s economy is mainly based on agribusiness.

The public mental health services network in the Ribeirão Preto catchment area is a regionalized system. Each community-based service is responsible for a specific catchment area regardless of the patient’s diagnosis or severity of the mental disorder. The inpatient units are closely connected with each other and with community-based services. When this cohort was started, the Ribeirão Preto catchment area had 108 psychiatric beds: 6 in an emergency hospital, 22 beds in psychiatric wards in a general hospital, and 80 beds in a psychiatric hospital.

### Study design

This is a longitudinal study based on medical records of a cohort of psychiatric patients (described in [20]) admitted between 2002 and 2007 to the public network of mental health services in the Ribeirão Preto catchment area. The cohort data were linked to the SEADE Foundation database (state data analysis system of São Paulo—https://www.seade.gov.br/), and the date and causes of death were collected. The SEADE Foundation uses deterministic linkage methods between patient records and death records (Supplementary Fig. S1). The outcome was deaths that occurred in the state of São Paulo from Jan 1, 2002 to Dec 31, 2016. This study followed the RECORD guidelines for reporting observational studies (Supplementary Table S1).

### Inclusion and exclusion criteria

We included all patients admitted for the first time to psychiatric beds distributed in three inpatient units (psychiatric hospital: 80 beds; general hospital: 22 beds; emergency unit: 6 beds) of the Ribeirão Preto catchment area from Jan 1, 2002 to Dec 31, 2007. First-admitted patients were identified in administrative databases provided by each inpatient unit, which were combined into a single database. We included patients who remained hospitalised for more than 12 h independent of the diagnoses.

Patients whose information regarding date of birth or mother's name was missing in the original database were excluded, as it prevented the identification of the individuals in the SEADE Foundation database where information on date and causes of death could be collected.

### Data collection

The beginning of the follow-up was considered the date of the first psychiatric admission, and the end of the follow-up was the date of death informed in the death certificate or Dec 31, 2016. The information available in the database obtained at the first hospital admission includes (a) sociodemographic data, including sex, age, occupation (employed/homemaker/student; unemployed), marital status (single/divorced/widowed; married/partnered), and city of origin (Ribeirão Preto; other municipalities); (b) psychiatric diagnosis according to Section V of the 10th edition of the International Classification of Diseases (ICD-10), including alcohol-related disorders (F10), nonalcohol psychoactive substance use disorders (F11–F19), psychotic disorders (F20–F29), mood disorders (F30–F39) and other mental disorders (F00–F09, F40–F48, F50–F59, F60–F69, F70–F79, F80–F89, and F90–F98 and F99); and (c) characteristics of hospital admission, including length of stay (in days), year of admission (2002–2007) and inpatient unit (psychiatric hospital, emergency unit, and general hospital). Regarding occupation and marital status, data were missing in 75 (1.86%) and 97 (2.41%) patients, respectively. To optimise our sample size, we imputed missing data using the Multiple Imputation by Chained Equations (MICE). The information available in the database obtained on the death certificate includes (a) sociodemographic data, including sex, age, skin colour (white; black/mixed/yellow) and marital status (single/divorced/widowed; married/partnered); and (b) information regarding death, including date, municipality of death (Ribeirão Preto; other municipalities), and cause of death according to the ICD-10, including natural causes (A00-R99) and unnatural causes (V01-Y98).

### Statistical analysis

We first performed a descriptive statistical analysis of the sample and follow-up data. We then estimated mortality rates (MRs) per 1000 person-years with 95% confidence intervals (95% CIs) for each exposure category. We estimated standardised mortality ratios (SMRs) with 95% CIs, adjusted for age group (10–14, 15–19, 20–29, 30–39, 40–49, 50–59, 60–69, 70–79, 80 +) and sex. We used the indirect method of standardisation, and we used data from the population of Ribeirão Preto from the last demographic census of 2010 (mid-follow-up period) as a standard population. In this reference population, the number of deaths for that period was 3,915 in 604,682 inhabitants (data obtained from the Brazilian Census Bureau—https://www.ibge.gov.br/). We calculated the life expectancy according to Chiang's method of abridged life tables with age intervals of 5 years [21]. Life expectancy was calculated for the entire cohort based on sex and diagnosis categories. The years of life lost (YLL) were calculated based on the difference between the age in death and expected age of death according to the annual actuarial life table from the SEADE Foundation for the period 2002–2016 divided by the number of deceased persons. The YLL reflects the years of life that the patient would have lived if the corresponding life expectancy had been fulfilled. We calculated average YLLs with 95% CI for each sex and diagnosis category. Survival analyses were then conducted to identify characteristics associated with overall mortality using Cox proportional hazards regression models (univariate and multivariate). We calculated hazard ratios (HRs) with 95% CIs for the univariate and multivariate models. Model 1 was adjusted for all covariates, and Model 2 was adjusted only for significant covariates in Model 1 (*p* < 0.05). The proportional hazard assumption was analysed with Cox’s proportional hazards test based on Schoenfeld residuals and visual inspection of log–log plots. The association of sociodemographic characteristics registered in the death certificate with mortality from natural and unnatural causes was also calculated. A survival analysis from the first hospital admission to death or the end of the observation was also computed using Kaplan–Meier estimates, and the log-rank test was calculated. The significance level was set at 0.05. Statistical analyses were performed using R software version 3.6.3 (R Core Team) and SAS software version 9.4 for Windows (SAS Institute Inc., Cary, NC, USA).

## Results

### Cohort characteristics

From Jan 1, 2002 to Dec 31, 2007, 4812 individuals had their first admission in a psychiatric bed available in the Ribeirão Preto catchment area. Information regarding date of birth or mother’s name was not available in 793 records, which were excluded for not meeting the criteria required in the procedure of linkage to the death databases. Thus, 4019 patients were included in our sample (see Supplementary Table S2). The total number of person-years in the study’s follow-up period was 42,720 (22,751 men and 19,969 women), and the median follow-up time of the cohort was 11.25 years. The median age of patients at first psychiatric admission was 35.00 years (interquartile range (IQR) 25.00–46.00). Eight hundred and three deaths (19.98%) were identified between Jan 01, 2002 and Dec 31, 2016, 560 in men and 243 in women. The median age of patients at death was 47.00 years (IQR 36.00–60.00) (Table 1).

**Table 1:** Characteristics of the cohort with first psychiatric admission from Jan 1, 2002 to Dec 31, 2007 and outcome of follow-up on Dec 31, 2016

	Women (*N* = 1818)	Men (*N* = 2201)	Total (*N* = 4019)
Cohort characteristics			
Person-years of follow-up	19,969	22,751	42,720
Median years of follow-up (IQR)	11.42 (9.92–12.92)	11.17 (9.33–12.87)	11.25 (9.67–12.92)
Median age (IQR)	35.00 (25.00–46.00)	35.00 (26.00–46.00)	35.00 (25.00–46.00)
Vital status on Dec 31, 2016			
Dead	243	560	803
Median age of dead (IQR)	51.00 (40.50–64.00)	45.00 (35.00–58.00)	47.00 (36.00–60.00)
Median years of follow-up of dead (IQR)	5.67 (2.58–9.17)	5.00 (2.14–8.50)	5.08 (2.25–8.67)
Time after admission until death; *N* (%)			
0–5 years	121 (49.79)	267 (47.68)	388 (48.32)
5–10 years	83 (34.16)	208 (37.14)	291 (36.24)
10–15 years	39 (16.05)	85 (15.18)	124 (15.44)

*IQR* interquartile range

The causes of death were divided into 18 categories (Table 2). Greater than 83% of deaths were related to natural causes, such as circulatory (19.18%), digestive (12.83%), and respiratory (12.70%) diseases. Patients admitted for alcohol-related disorders (F10) had a higher percentage of deaths related to diseases of the digestive system (K00–K95). In contrast, those admitted for nonalcohol psychoactive substance use (F11–F19) had deaths mainly related to external causes (V01–Y09), such as accidents, assaults and suicides. Psychotic disorders (F20–F29) had a higher percentage of deaths due to respiratory diseases (J00–J99), mood disorders (F30–F39) due to circulatory diseases (I00–I99), and other mental disorders (F00–F09 and F40–F99) due to endocrine, nutritional, metabolic (E00–E89) and neurological diseases (G00–G99).

**Table 2:** Causes of deaths in relation to the diagnosis at the first hospital admission by the International Classification of Diseases 10th version

Cause of death (ICD-10)	Diagnosis by ICD-10					
	F10	F11–F19	F20–F29	F30–F39	Others^a^	Total
	*N* (%)	*N* (%)	*N* (%)	*N* (%)	*N* (%)	*N* (%)
Natural						
Certain infectious and parasitic diseases (A00–B99)	21 (7.78)	7 (14.00)	6 (4.51)	14 (8.43)	11 (6.11)	59 (7.35)
Neoplasms (C00–D49)	29 (10.74)	4 (8.00)	12 (9.02)	15 (9.04)	19 (10.55)	79 (9.84)
Diseases of the blood and blood-forming organs and certain disorders involving the immune mechanism (D50–D89)	1 (0.37)	1 (2.00)	0 (0.00)	0 (0.00)	0 (0.00)	2 (0.25)
Endocrine, nutritional and metabolic diseases (E00–E89)	7 (2.59)	1 (2.00)	7 (5.26)	6 (3.61)	13 (7.22)	34 (4.23)
Mental, behavioural and neurodevelopmental disorders (F00–F99)	17 (6.30)	1 (2.00)	3 (2.26)	5 (3.01)	5 (2.78)	31 (3.86)
Diseases of the nervous system (G00–G99)	7 (2.59)	1 (2.00)	5 (3.76)	6 (3.61)	9 (5.00)	28 (3.49)
Diseases of the circulatory system (I00–I99)	42 (15.56)	6 (12.00)	24 (18.05)	42 (25.30)	39 (21.67)	154 (19.18)
Diseases of the respiratory system (J00–J99)	30 (11.11)	4 (8.00)	24 (18.05)	20 (12.05)	23 (12.77)	102 (12.70)
Diseases of the digestive system (K00–K95)	48 (17.78)	6 (12.00)	13 (9.77)	20 (12.05)	16 (8.89)	103 (12.83)
Diseases of the skin and subcutaneous tissue (L00–L99)	2 (0.74)	0 (0.00)	0 (0.00)	1 (0.60)	1 (0.56)	4 (0.50)
Diseases of the musculoskeletal system and connective tissue (M00–M99)	1 (0.37)	1 (2.00)	1 (0.75)	0 (0.00)	0 (0.00)	4 (0.50)
Diseases of the genitourinary system (N00–N99)	8 (2.96)	2 (4.00)	6 (4.51)	3 (1.81)	3 (1.67)	23 (2.86)
Congenital malformations, deformations and chromosomal abnormalities (Q00–Q99)	0 (0.00)	0 (0.00)	0 (0.00)	1 (0.60)	2 (1.11)	3 (0.37)
Symptoms, signs and abnormal clinical and laboratory findings, not elsewhere classified (R00–R99)	18 (6.67)	2 (4.00)	5 (3.76)	9 (5.42)	9 (5.00)	43 (5.35)
Unnatural						
Accidents (V01–X59)	19 (7.04)	6 (12.00)	10 (7.52)	10 (6.02)	12 (6.67)	57 (7.10)
Suicide (X60–X84)	11 (4.07)	5 (10.00)	4 (3.01)	7 (4.22)	4 (2.22)	31 (3.86)
Assault (X85–Y09)	3 (1.11)	2 (4.00)	5 (3.76)	2 (1.20)	5 (2.78)	17 (2.12)
Other external causes of morbidity and mortality (Y10–Y98)	6 (2.22)	1 (2.00)	8 (6.01)	5 (3.01)	9 (5.00)	29 (3.61)
Total	270 (100.00)	50 (100.00)	133 (100.00)	166 (100.00)	180 (100.00)	803 (100.00)

^a^Others mental disorders = F00–F09 and F40–F99; Alcohol-related disorders = F10; Nonalcohol psychoactive substance use = F11–F19; Psychotic disorders = F20–F29; Mood disorders = F30–F39

Thirty-one (3.86%) suicides were recorded (MR = 72.50/100,000 person-years; SMR = 11.41 95% CI 9.19–14.11), 20 in men (MR = 87.93/100,000 person-years; SMR = 8.14, 95% CI 6.62–9.81) and 11 in women (MR = 55.11/100,000 person-years; SMR = 27.00, 95% CI 21.09–34.48). The median age was 39 years (men = 36.50 years and women = 41 years). Regarding the diagnosis at the first admission, suicides occurred in 11 (35.48%) patients with alcohol use disorders (F10), 7 (22.58%) with mood disorders (F30–F39), 5 (16.13%) with nonalcohol psychoactive substance use disorders (F11–F19), 4 (12.90%) with psychotic disorders (F20–F29), and 4 (12.90%) with other mental disorders (F00–F09 and F40–F99).

### Mortality rates

Table 3 shows the overall mortality rates per 1000 person-years by sex and diagnosis. Overall, the risk of death was approximately three-fold (SMR = 2.90, 95% CI 2.71–3.11) in the general population with SMRs of 3.35 for men and 2.15 for women. The excess of mortality related to alcohol-related disorders (F10) was particularly high, i.e., increased five-fold compared with the rate for the reference population. Rates higher than expected were also observed for patients hospitalised due to the use of other psychoactive substances (F11–F19), psychotic disorders (F20–F29) and mood disorders (F30–F39).

**Table 3:** Mortality rates, standardised mortality ratios, excess mortality and years of life lost were reported in the cohort with the follow-up outcome in 2016

Diagnosis (ICD-10)	Sex	Person-year	MR (95% CI)	Deaths		Excess	SMR (95% CI)	YLL	Average YLL (95% CI)	Life expectancy (years)
				Observed	Expected	Mortality				
Alcohol-related disorders (F10)	Women	758	26.39 (16.57; 40.03)	20	4.07	15.93	4.91 (3.08; 7.45)	610.71	30.54 (26.27; 34.81)	47.98
	Men	6052	41.31 (36.42; 46.68)	250	44.59	205.41	5.60 (4.94; 6.33)	7,495.38	29.98 (28.84; 31.11)	41.32
	All	6810	39.65 (35.13; 44.59)	270	49.04	220.96	5.50 (4.87; 6.19)	8,106.09	30.02 (28.92; 31.12)	44.89
Nonalcohol psychoactive substance use (F11–F19)	Women	505	13.86 (6.06; 27.42)	7	2.84	4.16	2.46 (1.08; 4.87)	312.61	44.66 (34.25; 55.07)	33.86
	Men	2885	14.90 (10.92; 19.89)	43	21.26	21.74	2.02 (1.48; 2.70)	1,648.15	38.33 (34.66; 41.99)	32.97
	All	3390	14.75 (11.06; 19.29)	50	21.93	28.07	2.28 (1.71; 2.98)	1,960.76	39.22 (35.73; 42.71)	35.69
Psychotic disorders (F20–F29)	Women	3631	14.87 (11.28; 19.26)	54	20.47	33.53	2.64 (2.01; 3.41)	1,441.19	26.69 (24.02; 29.35)	51.83
	Men	5412	14.60 (11.63; 18.10)	79	39.88	39.12	1.98 (1.58; 2.46)	2,009.11	25.43 (22.74; 28.11)	45.87
	All	9043	14.71 (12.36; 17.37)	133	58.49	74.51	2.27 (1.91; 2.68)	3,450.30	25.94 (24.02; 27.86)	48.97
Mood disorders (F30–F39)	Women	9162	10.04 (8.14; 12.26)	92	51.65	40.35	1.78 (1.44; 2.17)	2,194.78	23.86 (21.45; 26.27)	54.66
	Men	4797	15.43 (12.20; 19.26)	74	35.35	38.65	2.09 (1.66; 2.61)	1,898.84	25.66 (22.64; 28.68)	45.64
	All	13,959	11.89 (10.18; 13.81)	166	90.29	75.71	1.84 (1.57; 2.13)	4,093.62	24.66 (22.78; 26.54)	50.25
Others mental disorders (F00–F09 and F40–F99)	Women	5913	11.84 (9.29; 14.87)	70	33.32	36.68	2.10 (1.65; 2.64)	2,033.87	29.06 (25.10; 33.02)	49.46
	Men	3605	31.62 (26.21; 37.84)	114	26.55	87.45	4.29 (3.55; 5.14)	2,557.37	22.43 (19.84; 25.02)	48.87
	All	9518	19.33 (16.69; 22.28)	184	61.54	122.46	2.99 (2.58; 3.44)	4,591.24	24.95 (22.71; 27.18)	49.96
All mental disorders (F00-F99)	Women	19,969	12.17 (10.71; 13.77)	243	113.17	129.83	2.15 (1.89; 2.43)	6,593.16	27.13 (25.42; 28.83)	51.39
	Men	22,751	24.61 (22.64; 26.72)	560	167.28	392.72	3.35 (3.08; 3.63)	15,608.85	27.87 (26.85; 28.89)	43.43
	All	42,720	18.80 (17.53; 20.13)	803	276.69	526.31	2.90 (2.71; 3.11)	22,202.01	27.64 (26.76; 28.52)	47.27

*MR* mortality rate per 1000 person-years; *SMR* standardised mortality ratio adjusted for sex and age; *YLL* years of life lost; life expectancy at birth in the state of São Paulo in 2010; *95% CI* 95% confidence interval; *ICD-10* International Classification of Diseases-10th revision

The 803 deceased persons lost more than 22 thousand years of life; the average YLL was 27.64 years. The YLL for men was higher (27.87 years) than that for women (27.13 years). In the sample with nonalcohol psychoactive substance-related disorders, YLL was higher (39.22 years) than that noted in other mental disorders, and women lost more years of life (44.66 years) than men (38.33 years). The life expectancy at birth in this cohort was 47.27 years.

According to Kaplan–Meier curves (see Supplementary Figure S2), the survival of men in our cohort was significantly lower than that of women (log-rank test: *p* < 0.01), and the survival of patients with alcohol-related disorders was significantly lower than that of patients with other diagnoses (log-rank test: *p* < 0.01). During the 15-year follow-up, the survival of patients admitted for alcohol use disorders was 57%; it is expected that the half-life, i.e., the estimated time that only 50% of the patients remain alive, was 17.21 years after the first psychiatric admission.

### Characteristics associated with a higher risk of death

In the univariate Cox regression analysis (Table 4), the risk of death was associated with sex (*p* < 0.01), age (*p* < 0.01), marital status (*p* = 0.03) and diagnosis (*p* < 0.01) at the first psychiatric admission. In a multivariate Cox regression analysis (Table 4) adjusted for all the variables (Model 1), mortality at the first psychiatric hospital admission was associated with sex (*p* < 0.01), age (*p* < 0.01), occupational status (*p* = 0.02) and diagnosis (*p* < 0.01). In model 2, a multivariate Cox regression analysis (Table 4) was adjusted exclusively for significant variables (*p* < 0.05), and higher mortality was associated with male sex (aHR = 1.62, 95% CI 1.37–1.92), age [20–39 years (aHR = 2.62, 95% CI 1.65–4.15), 40–59 years (aHR = 5.83, 95% CI 3.67–9.25) and ≥ 60 years (aHR = 21.47, 95% CI 13.48–34.17)], unemployment (aHR = 1.22, 95% CI 1.05–1.43), and diagnoses [alcohol-related disorders (aHR = 2.70, 95% CI 2.17–3.33), nonalcohol psychotics substance use (aHR = 1.79, 95% CI 1.27–2.53) and other mental disorders (aHR = 1.70, 95% CI 1.37–2.10)]. The proportional hazard assumption was analysed and confirmed. The global Schoenfeld test (*p* = 0.42) and visual inspection of curves log [-log (survival probability)] as a function of the follow-up time (in logarithmic scale) were considered (see Supplementary Figure S3). Cox regression models separately generated based on natural and unnatural causes of mortality are available in Supplementary Table S3.

**Table 4:** Survival analyses for univariate and multivariate Cox regression models for the variables at the first admission from all causes of mortality

	Cohort (*N* = 4019)	Death (*N* = 803)	Crude	Model 1	Model 2
	*N* (%)	*N* (%)	HR (95% CI)	aHR (95% CI)	aHR (95% CI)
Sex					
Women	1818 (45.23)	243 (30.26)	1.00	1.00	1.00
Men	2201 (54.77)	560 (69.74)	2.01 (**1.73; 2.34**)	1.95 (**1.66; 2.28**)	1.62 (**1.37; 1.92**)
Age group					
< 20 years	439 (10.92)	20 (2.49)	1.00	1.00	1.00
20–39 years	1990 (49.52)	245 (30.51)	2.82 (**1.79; 4.45**)	3.00 (**1.87; 4.82**)	2.62 (**1.65; 4.15**)
40–59 years	1279 (31.82)	334 (41.59)	6.48 (**4.13; 10.18**)	7.06 (**4.40; 11.32**)	5.83 (**3.67; 9.25**)
≥ 60 years	311 (7.74)	204 (25.41)	21.10 (**13.33; 33.41**)	21.90 (**13.58; 35.33**)	21.47 (**13.48; 34.17**)
Occupational status					
Employed/homemaker/student	1271 (31.62)	245 (30.51)	1.00	1.00	1.00
Unemployed	2748 (68.38)	558 (69.49)	1.04 (0.89; 1.21)	1.20 (**1.03; 1.41**)	1.22 (**1.05; 1.43**)
Marital status					
Single/divorced/widowed	2606 (64.84)	480 (59.78)	1.00	1.00	–
Married/partnered	1413 (35.16)	323 (40.22)	1.29 (**1.12; 1.49**)	0.97 (0.84; 1.13)	–
Hospital service					
Psychiatric hospital	1089 (27.09)	226 (28.15)	1.00	1.00	–
Emergency unit	2092 (52.05)	417 (51.93)	1.00 (0.85; 1.17)	1.19 (0.93; 1.54)	–
General hospital	838 (20.86)	160 (19.92)	0.90 (0.74; 1.11)	1.04 (0.84; 1.29)	–
Year of admission					
2002	610 (15.18)	157 (19.55)	1.00	1.00	–
2003	629 (15.65)	139 (17.31)	0.88 (0.70; 1.10)	0.82 (0.65; 1.03)	–
2004	779 (19.39)	154 (19.18)	0.81 (0.65; 1.01)	0.82 (0.65; 1.04)	–
2005	709 (17.64)	125 (15.57)	0.78 (**0.61; 0.99**)	0.75 (**0.59; 0.96**)	–
2006	675 (16.79)	118 (14.69)	0.85 (0.66; 1.08)	0.89 (0.69; 1.14)	-
2007	617 (15.35)	110 (13.70)	0.93 (0.72; 1.19)	1.04 (0.80; 1.35)	-
Length of stay					
1–2 days	1999 (49.75)	366 (45.58)	1.00	1.00	-
3–10 days	1064 (26.47)	240 (29.89)	1.23 (**1.04; 1.45**)	1.18 (0.99; 1.40)	-
11–30 days	652 (16.22)	136 (16.94)	1.09 (0.89; 1.33)	1.06 (0.86; 1.32)	-
31 days or more	304 (7.56)	61 (7.59)	1.06 (0.81; 1.39)	1.01 (0.76; 1.34)	-
Origin					
Other municipalities	1850 (46.03)	347 (43.21)	1.00	1.00	-
Ribeirão Preto	2169 (53.97)	456 (56.79)	1.12 (0.97; 1.29)	1.06 (0.91; 1.22)	-
Diagnosis (ICD-10)					
Mood disorders (F30–F39)	1263 (31.43)	166 (20.67)	1.00	1.00	1.00
Psychotic disorders (F20–F29)	823 (20.48)	133 (16.56)	1.24 (0.98; 1.55)	1.27 (**1.01; 1.62**)	1.29 (**1.02; 1.63**)
Nonalcohol psychoactive substance use (F11–F19)	319 (7.94)	50 (6.23)	1.26 (0.92; 1.73)	1.98 (**1.39; 2.82**)	1.79 (**1.27; 2.53**)
Alcohol-related disorders (F10)	714 (17.76)	270 (33.62)	3.36 (**2.77; 4.08**)	2.76 (**2.21; 3.45**)	2.70 (**2.17; 3.33**)
Others mental disorders^a^	900 (22.39)	184 (22.92)	1.62 (**1.32; 2.00**)	1.78 (**1.44; 2.22**)	1.70 (**1.37; 2.10**)

*HR* hazard ratio; *aHR* adjusted hazard ratio; model 1 include sex, age, occupational status, marital status, hospital service, year of admission, length of stay, origin and diagnosis; model 2 include sex, age, occupational status and diagnosis; *95% CI* 95% confidence interval; Bold significant values; *ICD-10* International Classification of Diseases-10th revision

^a^Others mental disorders = F00–F09 and F40–F99

The 803 deaths were categorised into natural causes (*N* = 669; 83.3%; A00–R99) and unnatural causes (*N* = 134; 16.7%; V01–Y98). In Table 5, unnatural causes of mortality were associated (*p* < 0.01) with age 16–39 years (41.04%), whereas natural causes are associated with the age of 60 years or older (38.72%). Although 74% of deaths occurred in patients with white skin colour, nonwhite patients died more often due to unnatural (32.84%) than natural (24.37%) causes (*p* = 0.03). Moreover, the diagnosis of nonalcohol psychoactive substance disorders at the first psychiatric admission increased the number of deaths by unnatural causes.

**Table 5:** Association of natural and unnatural causes of mortality for death certificate variables

	Natural (*N* = 669)	Unnatural (*N* = 134)	*p* value
	*N* (%)	*N* (%)	
Sex			0.20
Women	206 (30.79)	37 (27.61)	
Men	463 (69.21)	97 (72.39)	
Age group at death			<** 0.01**
16–39 years	103 (15.39)	55 (41.04)	
40–59 years	307 (45.89)	58 (43.29)	
≥ 60 years	259 (38.72)	21 (15.67)	
Skin colour			**0.03**
White	506 (75.63)	90 (67.16)	
Nonwhite	163 (24.37)	44 (32.84)	
Marital status at death			0.08
Married/partnered	190 (28.40)	28 (20.89)	
Single/divorced/widowed	479 (71.60)	106 (79.11)	
Municipality of death			0.50
Ribeirão Preto	350 (52.32)	65 (48.51)	
Other municipalities	319 (47.68)	69 (51.49)	
Diagnosis (ICD-10) in first admission			**0.01**
Mood disorders (F30–F39)	142 (21.22)	24 (17.91)	
Psychotic disorders (F20–F29)	106 (15.85)	27 (20.15)	
Nonalcohol psychoactive substance use (F11–F19)	36 (5.38)	14 (10.45)	
Alcohol-related disorders (F10)	231 (34.53)	39 (29.10)	
Others mental disorders (F00–F09 and F40–F99)	154 (23.02)	30 (22.39)	

*p* value = Chi-squared test; Bold significant values

*ICD-10* International Classification of Diseases-10th revision

### Sensitivity analysis

We used five cycles of multiple imputations via MICE. The multiple imputations used did not influence our results (see Supplementary Table S4); it only maximised the sample size, without directly influencing the results. The rate ratios of the sensitivity analysis were virtually the same throughout the study period compared to the initial results.

## Discussion

To the best of our knowledge, this is the first study performed in Latin America aiming to analyse the mortality of individuals after their first psychiatric admission, considering a wide range of diagnoses. Our results confirmed that people admitted to hospitals in the early stages of a psychiatric condition have an approximately three-fold greater risk of death than the general population, reducing the life expectancy by more than 27 years. Excess mortality was observed in all the diagnoses with a higher impact from alcohol and other psychoactive substance-related problems. We also observed that some sociodemographic features, such as sex and unemployment at the admission, may be associated with excess mortality in psychiatric patients.

The mortality rate in patients with mental disorders was 2.90-fold greater than that in the general population with an SMR ranging from 1.84 (95% CI 1.57–2.13) for mood disorders (F30–F39) to 5.50 (95% CI 4.87–6.19) for alcohol-related disorders (F10). All diagnoses were also associated with a shorter life expectancy with excess YLLs ranging from 24.66 for mood disorders to 39.22 for nonalcohol psychoactive substance use disorders. The pattern of overall excess mortality observed in our study is consistent with previous studies. However, the values were greater than those previously reported, mainly in HICs, which have a death risk of 2.22 and 10 YLLs [1]. Similarly, psychotic disorders were responsible for 25.94 (95% CI 24.02–27.86) YLLs in our study. This value was much higher than that reported in a systematic review [22] that covered all inhabited continents, except South America, where schizophrenia had a mean weight of 14.50 YLLs. This is a highly worrisome finding, considering that life expectancy at birth in LMICs is 13 years less than that in HICs (70.10 *vs.* 83.70) [23]. Several reasons can explain these discrepancies, such as social, political, and economic inequalities, urban violence and high suicide rates [24].

Our data are also consistent with previous studies indicating a reduction of more than 20 years in life expectancy in patients with alcohol use disorders [8, 9, 25]. However, we observed excess mortality in people with alcohol use disorders of greater than five times, which is higher than the values found in previous reports [26]. We also observed that the excess mortality from alcohol-related disorders was higher in men (SMR = 5.60, 95% CI 4.94–6.33) than in women (SMR = 4.91, 95% CI 3.08–7.45). This finding contrasts with the results of a meta-analysis [26] of 81 studies (mainly HICs), showing a greater risk of death for women (RR = 4.57, 95% CI 3.86–5.42) than men (RR = 3.38, 95% CI 2.98–3.84). A possible explanation is that in Brazil, the prevalence of heavy drinking is significantly lower in women than in men [27]. Thus, Brazilian women would be less likely to engage in problem drinking, develop alcohol-related disorders or alcohol withdrawal symptoms [28], and, as a consequence, have fewer health complications due to alcohol use.

We found a relatively low mortality rate in mood disorder patients (SMR = 1.84, 95% CI 1.57–2.13). This result differs from the only Brazilian study [19] conducted over 25 years ago, where excess mortality was concentrated in psychotic women who also presented depressive symptoms. This change over time has been previously reported [8, 29, 30] probably due to a reduction in social stigma on depression, which may have increased the treatment-seeking behaviour. In addition to a rapid diagnosis, adherence to drug treatment and monitoring by the mental health team may also have contributed to reducing mortality rates.

In general, more mortality due to unnatural causes was noted in patients who were admitted for disorders related to nonalcohol psychoactive substance use. This increase in mortality may be related to a higher risk of accidents with motor vehicles, the use of weapons and involvement in physical aggression and violence commonly associated with psychoactive substance use [31–33].

Notably, the mortality of psychiatric patients due to natural causes also remained higher than that of the reference population. Some studies attribute this increase in the association of schizophrenia with cardiovascular diseases [34, 35], depressive and neurotic disorders with gastrointestinal causes [5] and substance use disorders with liver diseases [36], highlighting the need for risk reduction, especially for these diseases. Excess mortality in patients with mental disorders has been associated with modifiable unhealthy lifestyles (e.g., smoking, poor diet, physical inactivity, alcohol use or drug use), which cause significant harm to health [35, 37, 38].

Our data also indicated that the highest excess mortality of psychiatric patients admitted for the first time occurred mainly in the first year after hospital admission, and approximately half of these deaths occurred less than 5 years after the first admission. Previous studies [39, 40] also reported that the highest mortality rate for psychiatric patients occurred in the first year after admission, especially deaths related to suicide [11, 39–41].

Although suicide mortality rates in our cohort have an SMR that is greater than 11 times higher than the rate for the Ribeirão Preto catchment area, our rates (72.50/100,000 person-years) were not as high as those reported in other studies (484.00/100,000 person-years) that integrated a meta-analysis [11] that investigated the suicide rate after discharge from psychiatric hospitalisation. This can be explained, at least partially, by the fact that Brazil is a country with strong religious beliefs since religiosity contributes as a protective factor in suicidal behaviour [42].

Our study also identified that individuals with nonwhite skin colour are more vulnerable to death from unnatural causes. This result differs from studies in HICs [43], showing higher mortality rates from unnatural causes in people with white skin colour. In Brazil, the excess of mortality due to unnatural causes in nonwhite skin colour may be related to the effects of inequalities, social disadvantages and discrimination [44, 45]. This information may help to develop targeted public health strategies in the future, but more in-depth studies in LMICs are needed.

### Strengths and limitations of the study

Our results should be considered given several limitations. First, the number of deaths may still be higher than what we reported. Our hospital admission data were linked in the databases of the SEADE Foundation (State data analysis system of São Paulo), which records only deaths that occurred in the state of São Paulo. We know that patients are mobile, and there may be deaths that occurred in other Brazilian states, which were not registered in our system. However, it does not diminish our findings since the mortality rates found are substantially large, highlighting the need for actions that must be taken to minimise them. Second, although our results can support a causal relationship between the diagnosis of mental disorders and mortality, caution is needed when interpreting these associations. Associations can also have a noncausal origin and result from confusion. Third, comorbidities and socioeconomic status are important potential confounding factors [38], which were not adjusted in this study due to a lack of information in the records. Fourth, difficulty in comparing the SMR with other countries with different age compositions and not standardised by the same reference population. Fifth, the number of patients included in our sample is not very large, and our results cover a single catchment area. Sixth, the study is also affected by possible errors and omissions that may exist in the databases, and data for some of the analysed variables are lacking.

Although our results need to be interpreted in the context of some limitations, our study is one of the few to assess associations between first psychiatric hospital admission, mortality and several psychiatric diagnoses. This is a strong point of our study because previous studies did not examine in detail the excess of alcohol-related mortality but the disorders due to substance use in general. Our study also contributes to the literature by exploring trends in excess mortality in a Brazilian cohort, using a population-based dataset covering a long period. In addition, these findings contribute substantially to a significant information gap from years of life lost in South America. Few studies have examined the mortality of psychiatric patients in population cohorts, especially in LMICs.

## Conclusion

The excess mortality reported in our study suggests that the first psychiatric hospitalisation reduces the life expectancy of people with mental disorders by 2–4 decades. Most patients died from potentially treatable diseases. Patients with severe mental disorders, especially those related to the use of alcohol and other psychoactive substances, should be a priority in preventive actions aimed at health. The study highlights the need for a broad and intensive follow-up in mental health services, adherence to treatment, and improvement of the patients’ physical health since the early stages of mental disorders.

## Supplementary Information

Below is the link to the electronic supplementary material.

Supplementary file1 (DOCX 15 KB)

Supplementary file2 (DOCX 22 KB)

Supplementary file3 (DOCX 39 KB)

Supplementary file4 (DOCX 23 KB)

Supplementary file5 (DOCX 56 KB)

Supplementary file6 (DOCX 163 KB)

Supplementary file7 (DOCX 499 KB)

## Data Availability

Data or any additional information is available upon request from the authors.

## References

[1] Walker ER, McGee RE, Druss BG (2015). Mortality in mental disorders and global disease burden implications: a systematic review and meta-analysis. JAMA Psychiat.

[2] Ajetunmobi O, Taylor M, Stockton D, Wood R (2013). Early death in those previously hospitalised for mental healthcare in Scotland: a nationwide cohort study, 1986–2010. BMJ Open.

[3] Toender A, Vestergaard M, Munk-Olsen T, Larsen JT, Kristensen JK, Laursen TM (2020). Risk of diabetic complications and subsequent mortality among individuals with schizophrenia and diabetes-a population-based register study. Schizophr Res.

[4] Keinänen J, Mantere O, Markkula N, Partti K, Perälä J, Saarni SI, Härkänenh T, Suvisaari J (2018). Mortality in people with psychotic disorders in Finland: a population-based 13-year follow-up study. Schizophr Res.

[5] Henriksson M, Nyberg J, Schiöler L, Hensing G, Kuhn GH, Söderberg M, Torén K, Löve J, Waern M, Aberg M (2018). Cause-specific mortality in Swedish males diagnosed with non-psychotic mental disorders in late adolescence: a prospective population-based study. J Epidemiol Community Health.

[6] Drake RE, Xie H, McHugo GJ (2020). A 16-year follow-up of patients with serious mental illness and co-occurring substance use disorder. World Psychiatry.

[7] Marmot M, Friel S, Bell R, Houweling TAJ, Taylor S (2008). Commission on social determinants of health. Closing the gap in a generation: health equity through action on the social determinants of health. Lancet.

[8] Carvalho AF, Heilig M, Perez A, Probst C, Rehm J (2019). Alcohol use disorders. Lancet.

[9] Westman J, Wahlbeck K, Laursen TM, Gissler M, Nordentoft M, Hallgren J, Arffman M, Osby U (2015). Mortality and life expectancy of people with alcohol use disorder in Denmark, Finland and Sweden. Acta Psychiatr Scand.

[10] Swaraj S, Wang M, Chung D, Curtis J, Firth J, Ramanuj PP, Sara G, Large M (2019). Meta-analysis of natural, unnatural and cause-specific mortality rates following discharge from in-patient psychiatric facilities. Acta Psychiatr Scand.

[11] Chung DT, Ryan CJ, Hadzi-Pavlovic D, Singh SP, Stanton C, Large MM (2017). Suicide rates after discharge from psychiatric facilities: a systematic review and meta-analysis. JAMA Psychiat.

[12] World Bank (2017). Atlas of sustainable development goals 2017: from world development indicators. World Bank Atlas.

[13] World Health Organization (2021) Noncommunicabe diseases. (https://www.who.int/news-room/fact-sheets/detail/noncommunicable-diseases). Accessed 19 Mar 2021

[14] Cohen A, Patel V, Thara R, Gureje O (2008). Questioning an axiom: better prognosis for schizophrenia in the developing world?. Schizophr Bull.

[15] Fekadu A, Medhin G, Kebede D, Alem A, Cleare AJ, Prince M, Hanlon C, Shibre T (2015). Excess mortality in severe mental illness: 10-year population-based cohort study in rural Ethiopia. Br J Psychiatry.

[16] Teferra S, Shibre T, Fekadu A, Medhin G, Wakwoya A, Alem A, Kullgren G, Jacobsson L (2011). (2011) Five-year mortality in a cohort of people with schizophrenia in Ethiopia. BMC Psychiatry.

[17] Park S, Kim SY, Hong JP (2015). Cause-specific mortality of psychiatric inpatients and outpatients in a general hospital in Korea. Asia Pacific Journal of Public Health.

[18] Mravcik V, Zabransky T, Talu A, Jasaitis E, Gafarova N, Musabekova Z (2014). Mortality of registered drug users in Central Asia. Int J Drug Policy.

[19] Menezes PR, Mann AH (1996). Mortality among patients with non-affective functional psychoses in a metropolitan area of south-eastern Brazil. Rev Saude Publica.

[20] Barros REM, Azevedo-Marques JM, Santos JLF, Zuardi AW, Del-Ben CM (2016). Impact of length of stay for first psychiatric admissions on the ratio of readmissions in subsequent years in a large Brazilian catchment area. Soc Psychiatry Psychiatr Epidemiol.

[21] Chiang CL (1979) Life table and mortality analysis. World Health Organisation. https://apps.who.int/iris/bitstream/handle/10665/62916/15736_eng.pdf

[22] Hjorthøj C, Stürup AE, McGrath JJ, Nordentoft M (2017). Years of potential life lost and life expectancy in schizophrenia: a systematic review and meta-analysis. Lancet Psychiatry.

[23] Kyu HH, Abate D, Abate KH, Abay SM, Abbafati C, Abbasi N (2018). Global, regional, and national disability-adjusted life-years (DALYs) for 359 diseases and injuries and healthy life expectancy (HALE) for 195 countries and territories, 1990–2017: a systematic analysis for the Global Burden of Disease Study 2017. Lancet.

[24] Fonseca L, Sena BF, Crossley N, Lopez-Jaramillo C, Koenen K, Freimer NB, Bressan RA, Belangero SI, Santoro ML, Gadelha A (2020). Diversity matters: opportunities in the study of the genetics of psychotic disorders in low-and middle-income countries in Latin America. Braz J Psychiatry.

[25] Esser MB, Sherk A, Liu Y, Naimi TS, Stockwell T, Stahre M, Kanny D, Landen M, Saitz R, Brewer RD (2020). Deaths and years of potential life lost from excessive alcohol use—United States, 2011–2015. Morb Mortal Wkly Rep.

[26] Roerecke M, Rehm J (2013). Alcohol use disorders and mortality: a systematic review and meta-analysis. Addiction.

[27] Garcia LP, Freitas LRSD (2015). Heavy drinking in Brazil: results from the 2013 National Health Survey. Epidemiol e Serv de Saúde.

[28] Erol A, Karpyak VM (2015). Sex and gender-related differences in alcohol use and its consequences: contemporary knowledge and future research considerations. Drug Alcohol Depend.

[29] Plana-Ripoll O, Pedersen CB, Agerbo E, Holtz Y, Erlangsen A, Canudas-Romo V (2019). A comprehensive analysis of mortality-related health metrics associated with mental disorders: a nationwide, register-based cohort study. Lancet.

[30] John U, Rumpf H, Hanke M, Meyer C (2020). Mental disorders and total mortality after 20 years in an adult general population sample. Eur Psychiatry.

[31] Callaghan RC, Gatley JM, Veldhuizen S, Lev-Ran S, Mann R, Asbridge M (2013). Alcohol-or drug-use disorders and motor vehicle accident mortality: a retrospective cohort study. Accid Anal Prev.

[32] Peacock A, Leung J, Larney S, Colledge S, Hickman M, Rehm J (2018). Global statistics on alcohol, tobacco and illicit drug use: 2017 status report. Addiction.

[33] Charlson FJ, Baxter AJ, Dua T, Degenhardt L, Whiteford HA, Vos T (2015). Excess mortality from mental, neurological and substance use disorders in the Global Burden of Disease Study 2010. Epidemiol Psychiatr Sci.

[34] Piotrowski P, Gondek TM, Królicka-Deręgowska A, Misiak B, Adamowski T, Kiejna A (2017). Causes of mortality in schizophrenia: an updated review of European studies. Psychiatr Danub.

[35] Moreno-Küstner B, Guzman-Parra J, Pardo Y, Sanchidrián Y, Díaz-Ruiz S, Mayoral-Cleries F (2021). Excess mortality in patients with schizophrenia spectrum disorders in Malaga (Spain): a cohort study. Epidemiol Psychiatr Sci.

[36] Nordentoft M, Wahlbeck K, Hällgren J, Westman J, Ösby U, Alinaghizadeh H (2013). Excess mortality, causes of death and life expectancy in 270,770 patients with recent onset of mental disorders in Denmark, Finland and Sweden. PLoS ONE.

[37] Liu NH, Daumit GL, Dua T, Aquila R, Charlson F, Cuijpers P, Druss B, Dudek K, Freeman M, Fujii C, Gaebel W (2017). Excess mortality in persons with severe mental disorders: a multilevel intervention framework and priorities for clinical practice, policy and research agendas. World Psychiatry.

[38] Haas AD, Ruffieux Y, van den Heuvel LL, Lund C, Boulle A, Euvrard J, Orrell C, Prozesky HW, Tiffin N, Lovero KL, Tlali M, Davies M-A, Wainberg ML (2020). Excess mortality associated with mental illness in people living with HIV in Cape Town, South Africa: a cohort study using linked electronic health records. Lancet Glob Health.

[39] Chung D, Hadzi-Pavlovic D, Wang M, Swaraj S, Olfson M, Large M (2019). Meta-analysis of suicide rates in the first week and the first month after psychiatric hospitalisation. BMJ Open.

[40] Large M, Swaraj S (2018). Suicide, substance use and natural causes are respectively the most important causes of mortality in the first year post discharge from psychiatric hospitals. Evid Based Ment Health.

[41] Kan CK, Ho TP, Dong JY, Dunn EL (2007). Risk factors for suicide in the immediate post-discharge period. Soc Psychiatry Psychiatr Epidemiol.

[42] Caribé AC, Nunez R, Montal D, Ribeiro L, Sarmento S, Quarantini LC, Miranda-Scippa Â (2012). Religiosity as a protective factor in suicidal behavior: a case-control study. J Nerv Ment Dis.

[43] Das-Munshi J, Chang CK, Dutta R, Morgan C, Nazroo J, Stewart R, Prince MJ (2017). Ethnicity and excess mortality in severe mental illness: a cohort study. Lancet Psychiatry.

[44] Pascoe EA, Smart RL (2009). Perceived discrimination and health: a meta-analytic review. Psychol Bull.

[45] Macinko J, Mullachery P, Proietti FA, Lima-Costa MF (2012). Who experiences discrimination in Brazil? Evidence from a large metropolitan region. Int J Equity Health.

