# Supplementary material for: Excess mortality in a cohort of Brazilian patients with a median follow-up of 11 years after the first psychiatric hospital admission
Source: Soc Psychiatry Psychiatr Epidemiol. 2022 May 31;58(2):319–30. doi: 10.1007/s00127-022-02304-z (PMC9922213; doi:10.1007/s00127-022-02304-z)
Supplement: Supplementary file 1 — Supplementary file1 (DOCX 15 KB) [file 127_2022_2304_MOESM1_ESM.docx]

**Supplementary Table S1.** Checklist of RECORD (REporting of studies Conducted using Observational Routinely-collected health Data) items.

|  | **RECORD Items** | **Reported in page** |
| --- | --- | --- |
| **Title and Abstract** | RECORD 1.1: The type of data used should be specified in the title or abstract. When possible, the name of the databases used should be included. | Page 2 |
|  | RECORD 1.2: If applicable, the geographic region and time frame within which the study took place should be reported in the title or abstract. | Page 2 |
|  | RECORD 1.3: If linkage between databases was conducted for the study, this should be clearly stated in the title or abstract. | Page 2 |
| **Methods** | RECORD 6.1: The methods of study population selection (such as codes or algorithms used to identify subjects) should be listed in detail. If this is not possible, an explanation should be provided. | Page 4 |
|  | RECORD 6.2: Any validation studies of the codes or algorithms used to select the population should be referenced. If validation was conducted for this study and not published elsewhere, detailed methods and results should be provided. | N/A |
|  | RECORD 6.3: If the study involved linkage of databases, consider use of a flow diagram or other graphical display to demonstrate the data linkage process, including the number of individuals with linked data at each stage. | Page 4 |
|  | RECORD 7.1: A complete list of codes and algorithms used to classify exposures, outcomes, confounders, and effect modifiers should be provided. If these cannot be reported, an explanation should be provided. | Page 5 |
|  | RECORD 12.1: Authors should describe the extent to which the investigators had access to the database population used to create the study population. | Page 4 |
|  | RECORD 12.2: Authors should provide information on the data cleaning methods used in the study. | Page 4 |
|  | RECORD 12.3: State whether the study included person-level, institutional-level, or other data linkage across two or more databases. The methods of linkage and methods of linkage quality evaluation should be provided. | Page 4 and Page 5 |
| **Results** | RECORD 13.1: Describe in detail the selection of the persons included in the study (i.e., study population selection), including filtering based on data quality, data availability, and linkage. The selection of included persons can be described in the text and/or by means of the study flow diagram. | Page 6 |
| **Discussion** | RECORD 19.1: Discuss the implications of using data that were not created or collected to answer the specific research question(s). Include discussion of misclassification bias, unmeasured confounding, missing data, and changing eligibility over time, as they pertain to the study being reported. | Page 10 |
| **Other Information** | RECORD 22.1: Authors should provide information on how to access any supplemental information such as the study protocol, raw data, or programming code. | Page 11 |

Reference: Benchimol EI, Smeeth L, Guttmann A, Harron K, Moher D, Petersen I, Sørensen HT, von Elm E, Langan SM, the RECORD Working Committee. The REporting of studies Conducted using Observational Routinely-collected health Data (RECORD) Statement. *PLoS Medicine, 12(10), e1001885, 2015.*
