# Supplementary material for: Excess mortality in a cohort of Brazilian patients with a median follow-up of 11 years after the first psychiatric hospital admission
Source: Soc Psychiatry Psychiatr Epidemiol. 2022 May 31;58(2):319–30. doi: 10.1007/s00127-022-02304-z (PMC9922213; doi:10.1007/s00127-022-02304-z)
Supplement: Supplementary file 2 — Supplementary file2 (DOCX 22 KB) [file 127_2022_2304_MOESM2_ESM.docx]

**Supplementary Table S2.** Characteristics of patients with first hospital admission included and excluded in the study.

|  | **Included (N=4,019)** | **Excluded (N=793)** |
| --- | --- | --- |
|  | **N (%)** | **N (%)** |
| **Sex** |  |  |
| Women | 1,818 (45.23) | 314 (39.59) |
| Men | 2,201 (54.77) | 479 (60.41) |
| **Age group** |  |  |
| < 20 years | 439 (10.93) | 55 (7.02) |
| 20 - 39 years | 1,990 (49.51) | 382 (48.79) |
| 40 - 59 years | 1,279 (31.82) | 0 (0.00) |
| ≥ 60 years | 311 (7.74) | 346 (44.19) |
| *Missing* | *0* | *10* |
| **Occupational status** |  |  |
| Employed/homemaker/student | 1,250 (31.69) | 34 (4.36) |
| Unemployed | 2,694 (68.31) | 745 (95.64) |
| *Missing* | *75* | *14* |
| **Marital status** |  |  |
| Single/divorced/widowed | 2,538 (64.71) | 288 (62.34) |
| Married/partnered | 1,384 (35.29) | 174 (37.66) |
| *Missing* | *97* | *331* |
| **Hospital Service** |  |  |
| Psychiatric hospital | 1,089 (27.09) | 62 (7.82) |
| Emergency unit | 2,092 (52.06) | 18 (2.27) |
| General hospital | 838 (20.85) | 713 (89.91) |
| **Year of admission** |  |  |
| 2002 | 610 (15.18) | 70 (8.83) |
| 2003 | 629 (15.65) | 111 (13.99) |
| 2004 | 779 (19.39) | 113 (14.25) |
| 2005 | 709 (17.64) | 119 (15.01) |
| 2006 | 675 (16.79) | 164 (20.68) |
| 2007 | 617 (15.35) | 216 (27.24) |
| **Length of stay** |  |  |
| 1–2 days | 1,999 (49.74) | 106 (13.37) |
| 3–10 days | 1,064 (26.47) | 291 (36.69) |
| 11–30 days | 652 (16.23) | 303 (38.21) |
| 31 days or more | 304 (7.56) | 93 (11.73) |
| **Origin** |  |  |
| Other municipalities | 1,850 (46.03) | 413 (52.08) |
| Ribeirão Preto | 2,169 (53.97) | 380 (47.92) |
| **Diagnosis (ICD-10)** |  |  |
| Mood disorders (F30-F39) | 1,263 (31.43) | 206 (25.98) |
| Psychotic disorders (F20-F29) | 823 (20.48) | 179 (22.57) |
| Nonalcohol psychoactive substance use (F11-F19) | 319 (7.94) | 103 (12.99) |
| Alcohol-related disorders (F10) | 714 (17.76) | 185 (23.33) |
| Others mental disorders (F00-F09 and F40-F99) | 900 (22.39) | 120 (15.13) |

ICD-10 = International Classification of Diseases - 10th revision
