# Supplementary material for: Excess mortality in a cohort of Brazilian patients with a median follow-up of 11 years after the first psychiatric hospital admission
Source: Soc Psychiatry Psychiatr Epidemiol. 2022 May 31;58(2):319–30. doi: 10.1007/s00127-022-02304-z (PMC9922213; doi:10.1007/s00127-022-02304-z)
Supplement: Supplementary file 3 — Supplementary file3 (DOCX 39 KB) [file 127_2022_2304_MOESM3_ESM.docx]

**Supplementary Table S3.** Survival analyses for univariate and multivariate Cox regression models for the variables at the first admission from unnatural and natural causes of mortality.

|  |  | **Unnatural** | | | | **Natural** | | | |
| --- | --- | --- | --- | --- | --- | --- | --- | --- | --- |
|  | **Cohort**  **(N=4,019)** | **Death**  **(N=134)** | **Crude** | **Model 1** | **Model 2** | **Death**  **(N=669)** | **Crude** | **Model 1** | **Model 2** |
|  | **N (%)** | **N (%)** | **HR (95% CI)** | **aHR (95% CI)** | **aHR (95% CI)** | **N (%)** | **HR (95% CI)** | **aHR (95% CI)** | **aHR (95% CI)** |
| **Sex** |  |  |  |  |  |  |  |  |  |
| Women | 1,818 (45.23) | 43 (32.09) | 1.00 | 1.00 | 1.00 | 200 (29.89) | 1.00 | 1.00 | 1.00 |
| Men | 2,201 (54.77) | 91 (67.91) | 1.99 (**1.38 ; 2.85**) | 1.75 (**1.16 ; 2.64**) | 1.61 (**1.07 ; 2.42**) | 469 (70.10) | 2.06 (**1.74 ; 2.42**) | 1.70 (**1.41 ; 2.05**) | 1.64 (**1.37 ; 1.98**) |
| **Age group** |  |  |  |  |  |  |  |  |  |
| < 20 years | 439 (10.92) | 3 (2.24) | 1.00 | 1.00 | 1.00 | 17 (2.54) | 1.00 | 1.00 | 1.00 |
| 20 - 39 years | 1,990 (49.52) | 56 (41.79) | 4.43 (**1.39 ; 14.17**) | 4.87 (**1.50 ; 15.81**) | 4.96 (**1.54 ; 15.97**) | 189 (28.25) | 2.58 (**1.57 ; 4.25**) | 2.58 (**1.53 ; 4.34**) | 2.45 (**1.49 ; 4.05**) |
| 40 - 59 years | 1,279 (31.82) | 48 (35.82) | 6.93 (**2.16 ; 22.27**) | 7.25 (**2.17 ; 24.26**) | 7.98 (**2.43 ; 26.24**) | 286 (42.75) | 6.63 (**4.06 ; 10.81**) | 6.33 (**3.76 ; 10.67**) | 5.88 (**3.57 ; 9.68**) |
| ≥ 60 years | 311 (7.74) | 27 (20.15) | 30.79 (**9.34 ; 101.52**) | 37.61 (**11.02 ; 128.37**) | 41.94 (**12.50 ; 140.71**) | 177 (26.46) | 22.95 (**13.95 ; 37.76**) | 23.88 (**14.09 ; 40.51**) | 22.99 (**13.89 ; 38.15**) |
| **Occupational status** |  |  |  |  |  |  |  |  |  |
| Employed/homemaker/student | 1,271 (31.62) | 26 (19.40) | 1.00 | 1.00 | 1.00 | 219 (32.73) | 1.00 | 1.00 | - |
| Unemployed | 2,748 (68.38) | 108 (80.60) | 1.89 (**1.23 ; 2.91**) | 2.20 (**1.40 ; 3.47**) | 2.10 (**1.34 ; 3.29**) | 450 (67.27) | 1.04 (0.81 ; 1.12) | 1.18 (0.99 ; 1.41) | - |
| **Marital status** |  |  |  |  |  |  |  |  |  |
| Single/divorced/widowed | 2,606 (64.84) | 79 (58.95) | 1.00 | 1.00 | - | 401 (59.94) | 1.00 | 1.00 | - |
| Married/partnered | 1,413 (35.16) | 55 (41.05) | 1.38 (0.98 ; 1.95) | 1.24 (0.86 ; 1.79) | - | 268 (40.06) | 1.29 (**1.10 ; 1.51**) | 0.97 (0.82 ; 1.14) | - |
| **Hospital Service** |  |  |  |  |  |  |  |  |  |
| Psychiatric hospital | 1,089 (27.09) | 47 (35.07) | 1.00 | 1.00 | - | 179 (26.76) | 1.00 | 1.00 | 1.00 |
| Emergency unit | 2,092 (52.05) | 60 (44.78) | 0.69 (0.47 ; 1.02) | 1.65 (0.78 ; 3.48) | - | 357 (53.36) | 1.09 (0.91 ; 1.30) | 1.91 (**1.38 ; 2.65**) | 1.81 (**1.32 ; 2.50**) |
| General hospital | 838 (20.86) | 27 (20.15) | 0.73 (0.46 ; 1.18) | 1.00 (0.59 ; 1.70) | - | 133 (19.88) | 0.98 (0.77 ; 1.19) | 1.28 (0.99 ; 1.65) | 1.18 (0.93 ; 1.51) |
| **Year of admission** |  |  |  |  |  |  |  |  |  |
| 2002 | 610 (15.18) | 30 (22.39) | 1.00 | 1.00 | 1.00 | 127 (18.98) | 1.00 | 1.00 | 1.00 |
| 2003 | 629 (15.66) | 24 (17.92) | 0.75 (0.44 ; 1.29) | 0.68 (0.39 ; 1.19) | 0.72 (0.42 ; 1.25) | 115 (17.19) | 0.93 (0.71 ; 1.19) | 0.90 (0.69 ; 1.17) | 0.91 (0.71 ; 1.18) |
| 2004 | 779 (19.38) | 18 (13.43) | 0.47 (**0.26 ; 0.84**) | 0.47 (**0.25 ; 0.86**) | 0.48 (**0.26 ; 0.89**) | 136 (20.33) | 0.92 (0.72 ; 1.18) | 0.90 (0.69 ; 1.17) | 0.94 (0.74 ; 1.21) |
| 2005 | 709 (17.64) | 23 (17.16) | 0.70 (0.40 ; 1.21) | 0.60 (0.34 ; 1.07) | 0.65 (0.37 ; 1.15) | 102 (15.25) | 0.83 (0.64 ; 1.08) | 0.75 (**0.56 ; 0.98**) | 0.79 (0.60 ; 1.04) |
| 2006 | 675 (16.79) | 23 (17.16) | 0.79 (0.46 ; 1.38) | 0.86 (0.49 ; 1.52) | 0.82 (0.47 ; 1.44) | 95 (14.20) | 0.89 (0.69 ; 1.19) | 0.94 (0.70 ; 1.25) | 1.02 (0.78 ; 1.36) |
| 2007 | 617 (15.35) | 16 (11.94) | 0.67 (0.36 ; 1.25) | 0.67 (0.35 ; 1.29) | 0.71 (0.37 ; 1.35) | 94 (14.05) | 1.05 (0.80 ; 1.39) | 1.13 (0.85 ; 1.52) | 1.22 (0.92 ; 1.62) |
| **Length of stay** |  |  |  |  |  |  |  |  |  |
| 1–2 days | 1,999 (49.74) | 51 (38.06) | 1.00 | 1.00 | 1.00 | 315 (47.08) | 1.00 | 1.00 | 1.00 |
| 3–10 days | 1,064 (26.47) | 47 (35.07) | 1.75 (**1.18 ; 2.60**) | 2.04 (**1.03 ; 4.04**) | 1.40 (0.92 ; 2.13) | 193 (28.85) | 1.15 (0.96 ; 1.37) | 1.60 (**1.20 ; 2.12**) | 1.61 (**1.22 ; 2.13**) |
| 11–30 days | 652 (16.23) | 25 (18.66) | 1.46 (0.90 ; 2.36) | 2.09 (0.89 ; 4.86) | 1.27 (0.77 ; 2.10) | 111 (16.59) | 1.06 (0.86 ; 1.28) | 1.74 (**1.20 ; 2.51**) | 1.79 (**1.24 ; 2.57**) |
| 31 days or more | 304 (7.56) | 11 (8.21) | 1.39 (0.72 ; 2.67) | 1.94 (0.74 ; 5.07) | 1.16 (0.58 ; 2.30) | 50 (7.48) | 1.00 (0.74 ; 1.35) | 1.59 (**1.04 ; 2.44**) | 1.61 (**1.05 ; 2.46**) |
| **Origin** |  |  |  |  |  |  |  |  |  |
| Other municipalities | 1,850 (46.03) | 58 (43.28) | 1.00 | 1.00 | - | 380 (56.80) | 1.00 | 1.00 | - |
| Ribeirão Preto | 2,169 (53.97) | 76 (56.72) | 1.14 (0.81 ; 1.61) | 1.19 (0.84 ; 1.69) | - | 289 (43.20) | 0.88 (0.76 ; 1.03) | 0.94 (0.80 ; 1.10) | - |
| **Diagnosis (ICD-10)** |  |  |  |  |  |  |  |  |  |
| F30-F39 | 1,263 (31.43) | 24 (17.91) | 1.00 | 1.00 | 1.00 | 142 (21.22) | 1.00 | 1.00 | 1.00 |
| F20-F29 | 823 (20.48) | 27 (20.15) | 1.75 (**1.01 ; 3.03**) | 1.82 (**1.02 ; 3.22**) | 1.78 (**1.01 ; 3.13**) | 106 (15.85) | 1.16 (0.90 ; 1.49) | 1.25 (0.96 ; 1.64) | 1.27 (0.98 ; 1.64) |
| F11-F19 | 319 (7.94) | 14 (10.45) | 2.41 (**1.24 ; 4.65**) | 2.80 (**1.31 ; 5.95**) | 2.80 (**1.33 ; 5.87**) | 36 (5.38) | 1.08 (0.75 ; 1.56) | 1.71 (**1.14 ; 2.56**) | 1.70 (**1.15 ; 2.51**) |
| F10 | 714 (17.76) | 39 (29.10) | 3.88 (**2.34 ; 6.46**) | 3.13 (**1.73 ; 5.65**) | 3.07 (**1.72 ; 5.45**) | 231 (34.53) | 3.40 (**2.76 ; 4.19**) | 2.94 (**2.30 ; 3.76**) | 2.74 (**2.16 ; 3.47**) |
| F00-F09 and F40-F99 | 900 (22.39) | 30 (22.39) | 1.89 (**1.10 ; 3.24**) | 2.15 (**1.24 ; 3.74**) | 2.13 (**1.23 ; 3.68**) | 154 (23.02) | 1.61 (**1.28 ; 2.02**) | 1.84 (**1.45 ; 2.34**) | 1.80 (**1.43 ; 2.28**) |

F30-F39 = Mood disorders; F20-F29 = Psychotic disorders; F11-F19 = Nonalcohol psychoactive substance use; F10 = Alcohol-related disorders; F00-F09 and F40-F99 = others mental disorders; HR= Hazard Ratio; aHR= adjusted Hazard Ratio; 95% CI= 95% Confidence Interval; Bold significant values; ICD-10 = International Classification of Diseases - 10th revision

Unnatural: Model 1 include sex, age, occupational status, marital status, hospital service, year of admission, length of stay, origin and diagnosis; Model 2 include sex, age, occupational status, year of admission, length of stay and diagnosis;

Natural: Model 1 include sex, age, occupational status, marital status, hospital service, year of admission, length of stay, origin and diagnosis; Model 2 include sex, age, hospital service, year of admission, length of stay and diagnosis.
