# Supplementary material for: Excess mortality in a cohort of Brazilian patients with a median follow-up of 11 years after the first psychiatric hospital admission
Source: Soc Psychiatry Psychiatr Epidemiol. 2022 May 31;58(2):319–30. doi: 10.1007/s00127-022-02304-z (PMC9922213; doi:10.1007/s00127-022-02304-z)
Supplement: Supplementary file 4 — Supplementary file4 (DOCX 23 KB) [file 127_2022_2304_MOESM4_ESM.docx]

**Supplementary Table S4.** Cox regression models for the variables without multiple imputations.

|  | **Cohort**  **(N=4,019)** | **Death**  **(N=803)** | **Crude** | **Model 1** | **Model 2** |
| --- | --- | --- | --- | --- | --- |
|  | **N (%)** | **N (%)** | **HR (95% CI)** | **aHR (95% CI)** | **aHR (95% CI)** |
| **Sex** |  |  |  |  |  |
| Women | 1,818 (45.23) | 243 (30.26) | 1.00 | 1.00 | 1.00 |
| Men | 2,201 (54.77) | 560 (69.74) | 2.01 (**1.73 ; 2.34**) | 2.07 (**1.74 ; 2.40**) | 1.61 (**1.37 ; 1.91**) |
| **Age** |  |  |  |  |  |
| < 20 years | 439 (10.92) | 20 (2.49) | 1.00 | 1.00 | 1.00 |
| 20 - 39 years | 1,990 (49.52) | 245 (30.51) | 2.82 (**1.79 ; 4.45**) | 2.86 (**1.79 ; 4.53**) | 2.73 (**1.72 ; 4.32**) |
| 40 - 59 years | 1,279 (31.82) | 334 (41.60) | 6.48 (**4.13 ; 10.18**) | 6.22 (**3.89 ; 9.91**) | 6.07 (**3.83 ; 9.63**) |
| ≥ 60 years | 311 (7.74) | 204 (25.40) | 21.10 (**13.33 ; 33.41**) | 21.72 (**13.55 ; 34.79**) | 21.36 (**13.42 ; 34.01**) |
| **Occupational status** |  |  |  |  |  |
| Employed/homemaker/student | 1,250 (31.10) | 243 (30.26) | 1.00 | 1.00 | 1.00 |
| Unemployed | 2,694 (67.03) | 544 (67.75) | 1.06 (0.91 ; 1.24) | 1.26 (**1.07 ; 1.48**) | 1.24 (**1.06 ; 1.45**) |
| *Missing* | 75 (1.87) | 16 (1.99) |  |  |  |
| **Marital status** |  |  |  |  |  |
| Single/divorced/widowed | 2,538 (63.16) | 465 (57.90) | 1.00 | 1.00 | - |
| Married/partnered | 1,384 (34.43) | 319 (39.72) | 1.27 (**1.11 ; 1.47**) | 0.98 (0.85 ; 1.14) | - |
| *Missing* | 97 (2.41) | 19 (2.37) |  |  |  |
| **Hospital Service** |  |  |  |  |  |
| Psychiatric hospital | 1,089 (27.09) | 226 (28.15) | 1.00 | 1.00 | - |
| Emergency unit | 2,092 (52.05) | 417 (51.93) | 1.00 (0.85 ; 1.17) | 1.08 (0.96 ; 1.24) | - |
| General hospital | 838 (20.86) | 160 (19.92) | 0.90 (0.74 ; 1.11) | 1.07 (0.94 ; 1.26) | - |
| **Year of admission** |  |  |  |  |  |
| 2002 | 610 (15.18) | 157 (19.55) | 1.00 | 1.00 | - |
| 2003 | 629 (15.65) | 139 (17.31) | 0.88 (0.70 ; 1.10) | 0.87 (0.69 ; 1.10) | - |
| 2004 | 779 (19.39) | 154 (19.18) | 0.81 (0.65 ; 1.01) | 0.90 (0.71 ; 1.13) | - |
| 2005 | 709 (17.64) | 125 (15.57) | 0.78 (**0.61 ; 0.99**) | 0.77 (**0.60 ; 0.98**) | - |
| 2006 | 675 (16.79) | 118 (14.69) | 0.85 (0.66 ; 1.08) | 1.00 (0.80 ; 1.20) | - |
| 2007 | 617 (15.35) | 110 (13.70) | 0.93 (0.72 ; 1.19) | 1.05 (0.79 ; 1.32) | - |
| **Length of stay** |  |  |  |  |  |
| 1–2 days | 1,999 (49.75) | 366 (45.58) | 1.00 | 1.00 | - |
| 3–10 days | 1,064 (26.47) | 240 (29.89) | 1.23 (**1.04 ; 1.45**) | 1.21 (0.99 ; 1.42) | - |
| 11–30 days | 652 (16.22) | 136 (16.94) | 1.09 (0.89 ; 1.33) | 1.08 (0.89 ; 1.32) | - |
| 31 days or more | 304 (7.56) | 61 (7.59) | 1.06 (0.81 ; 1.39) | 1.05 (0.80 ; 1.37) | - |
| **Origin** |  |  |  |  |  |
| Other municipalities | 1,850 (46.03) | 347 (43.21) | 1.00 | 1.00 | - |
| Ribeirão Preto | 2,169 (53.97) | 456 (56.79) | 1.12 (0.97 ; 1.29) | 0.93 (0.81 ; 1.07) | - |
| **Diagnosis (ICD-10)** |  |  |  |  |  |
| Mood disorders (F30-F39) | 1,263 (31.43) | 166 (20.67) | 1.00 | 1.00 | 1.00 |
| Psychotic disorders (F20-F29) | 823 (20.48) | 133 (16.56) | 1.24 (0.98 ; 1.55) | 1.27 (**1.01 ; 1.61**) | 1.28 (**1.02 ; 1.62**) |
| Nonalcohol psychoactive (F11-F19) | 319 (7.94) | 50 (6.23) | 1.26 (0.92 ; 1.73) | 1.85 (**1.32 ; 2.61**) | 1.87 (**1.34 ; 2.61**) |
| Alcohol-related disorders (F10) | 714 (17.76) | 270 (33.62) | 3.36 (**2.77 ; 4.08**) | 2.75 (**2.21 ; 3.44**) | 2.67 (**2.16 ; 3.30**) |
| Others mental disorders* | 900 (22.39) | 184 (22.92) | 1.62 (**1.32 ; 2.00**) | 1.71 (**1.38 ; 2.13**) | 1.71 (**1.38 ; 2.11**) |

*Others mental disorders= F00-F09 and F40-F99; HR= Hazard Ratio; aHR= adjusted Hazard Ratio; Model 1 include sex, age, occupational status, marital status, hospital service, year of admission, length of stay, origin and diagnosis; Model 2 include sex, age, occupational status and diagnosis; 95% CI= 95% Confidence Interval; Bold significant values; ICD-10 = International Classification of Diseases - 10th revision
