# Supplementary figures and images for: Excess mortality in a cohort of Brazilian patients with a median follow-up of 11 years after the first psychiatric hospital admission
Source: Soc Psychiatry Psychiatr Epidemiol. 2022 May 31;58(2):319–30. doi: 10.1007/s00127-022-02304-z (PMC9922213; doi:10.1007/s00127-022-02304-z)

**Supplementary Figure S1.** Flow Diagram for cohort characteristics and linked data at each stage.


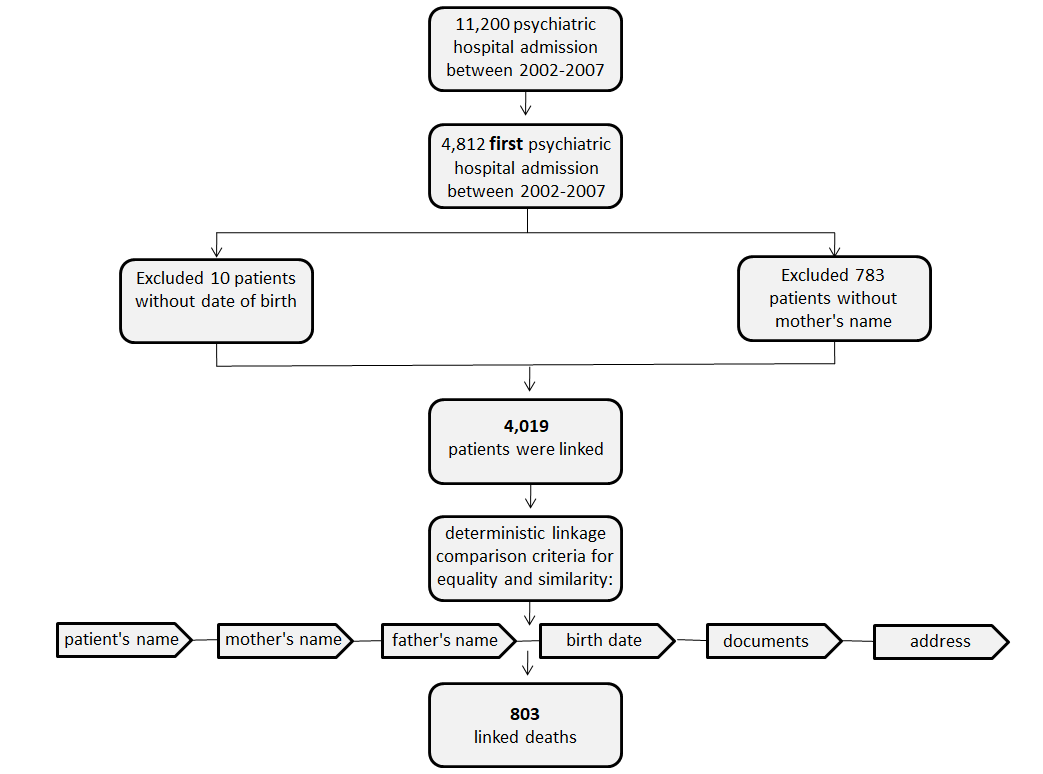

Supplement: Supplementary file 5 — Supplementary file5 (DOCX 56 KB) [file 127_2022_2304_MOESM5_ESM.docx]

**Supplementary Figure S2.** Survival analysis **(**Kaplan-Meier curves) by sex and diagnosis.


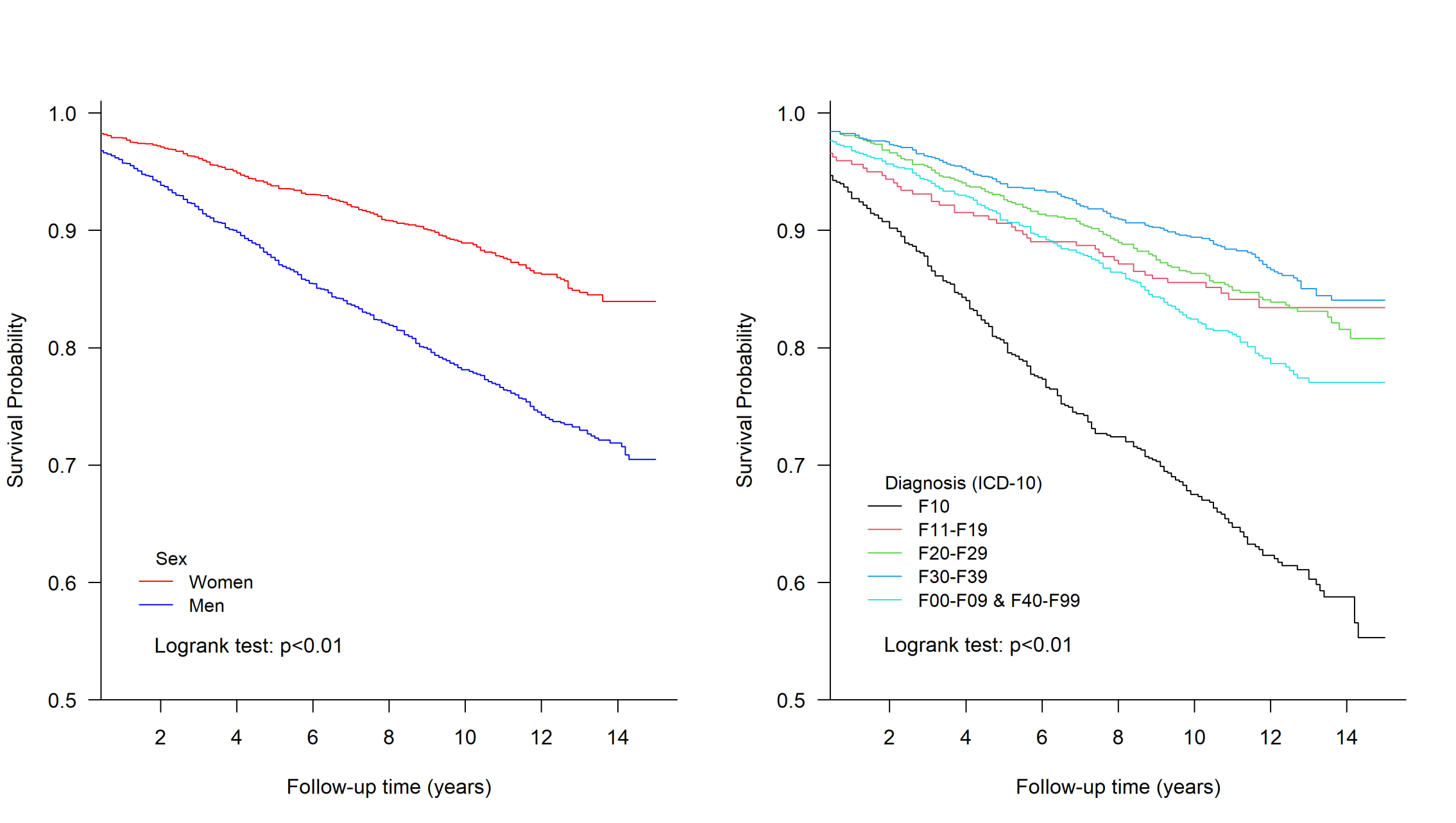

Supplement: Supplementary file 6 — Supplementary file6 (DOCX 163 KB) [file 127_2022_2304_MOESM6_ESM.docx]
