# Supplementary material for: Excess mortality in a cohort of Brazilian patients with a median follow-up of 11 years after the first psychiatric hospital admission
Source: Soc Psychiatry Psychiatr Epidemiol. 2022 May 31;58(2):319–30. doi: 10.1007/s00127-022-02304-z (PMC9922213; doi:10.1007/s00127-022-02304-z)
Supplement: Supplementary file 7 — Supplementary file7 (DOCX 499 KB) [file 127_2022_2304_MOESM7_ESM.docx]

**Supplementary Figure S3.** Proportional hazard functions. Curves log (-log (survival probability)) as a function of the follow-up time (logarithmic scale) for sex (A), age (B), occupational status (C) and diagnosis (D).

| (A) | (B) |
| --- | --- |
| 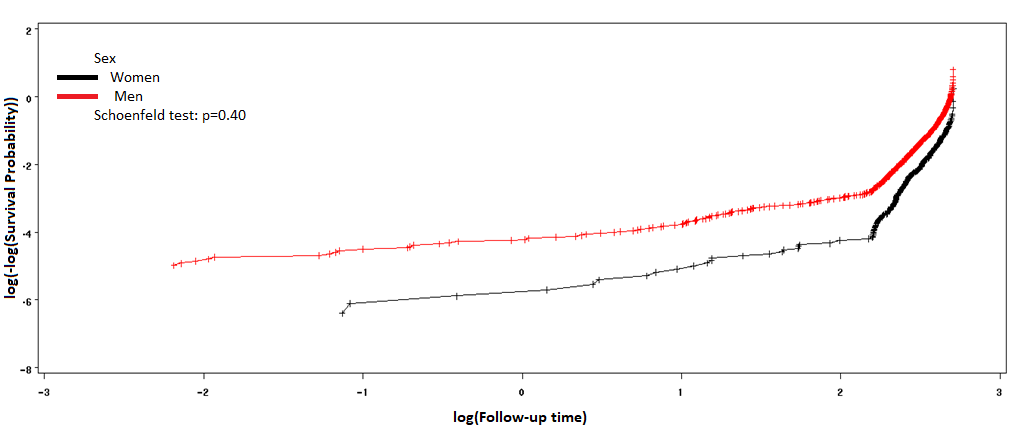 | 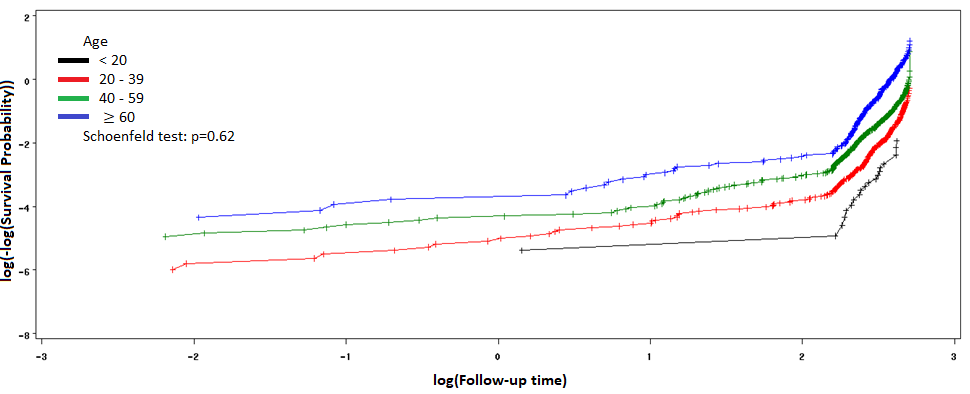 |
| (C) | (D) |
| 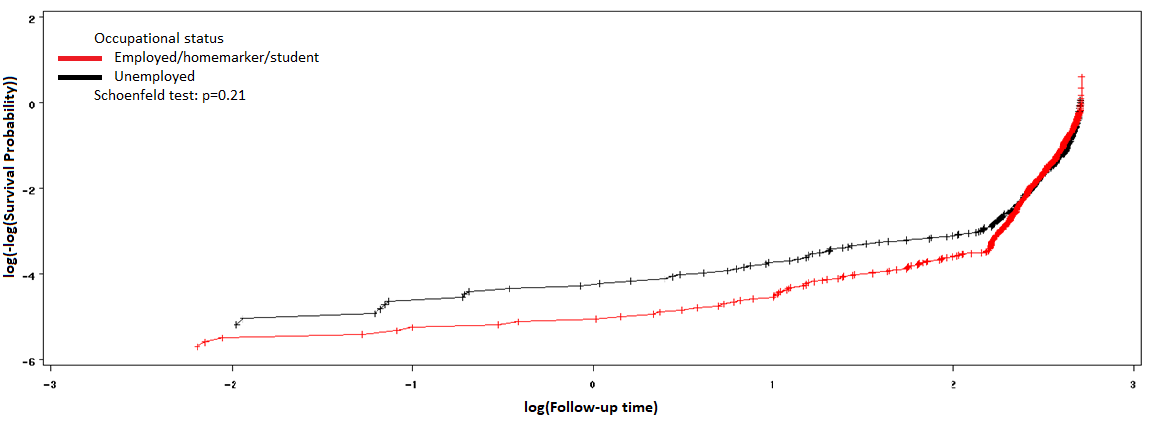 | 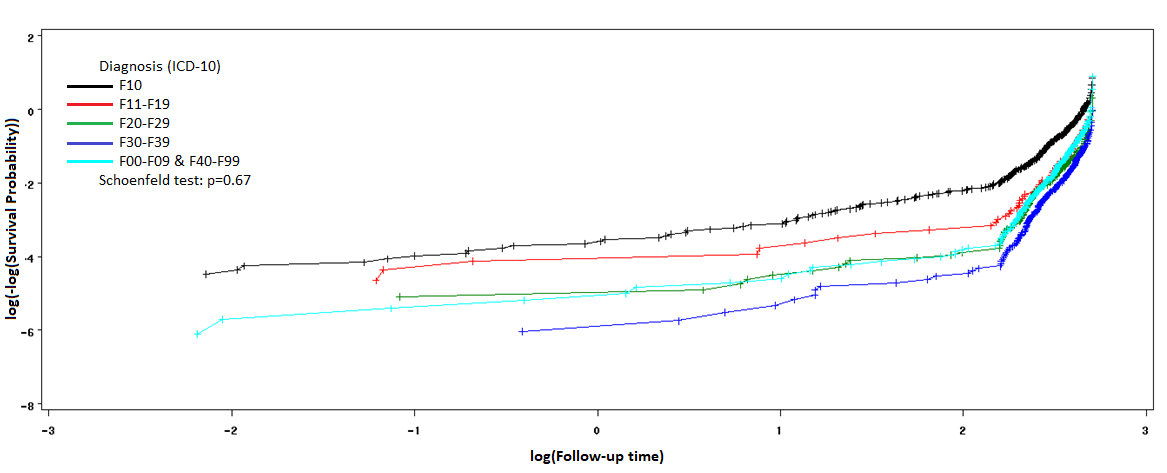 |
| Global Schoenfeld test: p= 0.42 | |
